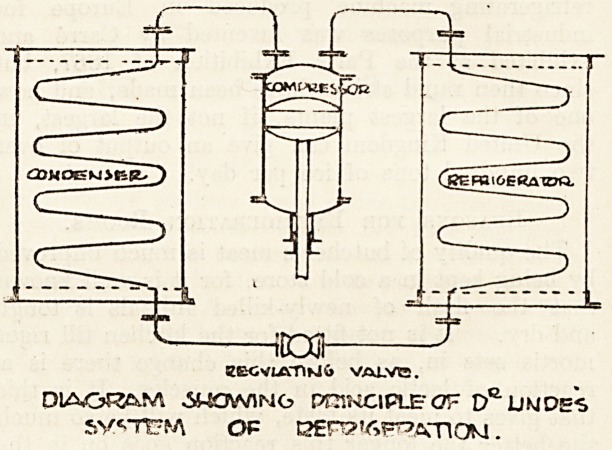# Cold-Storage Problems

**Published:** 1911-04-15

**Authors:** 


### Hospital Architecture and Construction.

[Communications on this subject should be marked "Architecture" in the left-hand top corner of the enve

### COLD-STORAGE PROBLEMS.

### I.—ICE *v.* MECHANICAL REFRIGERATION.

One of the most important auxiliaries to the modern hospital and kindred establishments is the Cold Store and its subordinates. By their aid the necessary animal and vegetable foods may be kept in proper condition and a cold temperature may be obtained for pathological purposes, pasteurising, etc. The advantages which a cold store offer are so evident and affect the most varied interests so much, that it actually appears incredible that a hospital establishment of any size should be built without one in the present times. The first refrigerating machine produced in Europe for industrial purposes was invented by Carré and exhibited at the Paris Exhibition of 1867, but since then rapid strides have been made, and now one of the largest plants, if not the largest, in the United Kingdom can give an output of over two hundred tons of ice per day.

Reasons for Refrigeration Rooms.

The quality of butcher's meat is much improved by being kept in a cold store, for it is well known that the flesh of newly-killed animals is tough and dry. It is not fitted for the kitchen till rigor mortis sets in, as before this change there is a reaction of lactic acid in the muscles. It is this that gives to meat its taste, which will be so much the better the longer this reaction goes on in the muscles and the connecting tissues. This condition can be attained artificially by laying the meat in wine, vinegar, or sour milk. This ripening process can go on in the cold store while at the same time the meat is kept under conditions unfavourable to putrefaction, and anything which will lead to it, for it is well known that the keeping of meat in cold and at the same time dry and fresh air is the best method of meat preservation. It is not only better for culinary purposes but for human health as it excludes every possible danger of contamination.

Some General Considerations.

Rooms cooled by ice are very useful if sufficient ventilation is provided, but this condition is seldom arranged for, owing to the generally prevailing idea that the ice will melt too rapidly, while no consideration is given to the fact that by the melting of the ice the air becomes strongly saturated with moisture which has a deleterious effect on the meat. To carry off this air, and replace it by dry air, ventilating appliances have been devised whereby a rapid change of air is possible, thus maintaining both a cold and a dry atmosphere. In some cases this method acts satisfactorily, but it is not a popular one. The temperature is always over 32° F.; indeed on account of the air brought in with the meat it may rise to 38° or 40° F. This tends to produce super-saturation which will cause condensation on the walls and roof, and possibly on already cooled meat this providing a favourable propagating ground for bacteria. Thirty-seven thousand cubic feet should not contain more than nine pounds of aqueous vapour; it is therefore essential that the air be cooled to between 30° and 28° F. before any moisture can be deposited, because at this temperature it contains exactly the quantity of water required to bring it to a saturated condition. To cool the air to such a low temperature with lumps of ice seems impossible, and therefore we must conclude that cooling by means of ice does not meet the demand for a satisfactory refrigerator.

Indeed natural ice has been long condemned as an agent for preserving foods any length of time. Different classes of food require different temperatures, which cannot be secured by the use of ice, but which can be perfectly controlled by mechanical refrigeration.

Mechanical Refrigeration.

The advantages of mechanical refrigeration are becoming better known and appreciated, and it has already become indispensable to many industries, such as brewing, meat importation, bacon curing, chocolate cooling, chemical manufacture, mining, oil refining, sugar refining, and other purposes too numerous to mention.

The process further supplies pure hygienic ice at all seasons and does away with the danger from the poisonous germs which thrive in wood and metal lined cold closets that become water-logged from melting ice.

The necessary temperature of cold rooms varies for different purposes, as the following list will demonstrate, but cleanliness, dryness, thorough ventilation and pure air are essential in each case. Periodical disinfection with formaldehyde gas is efficacious:—

| | Deg. Deg. |
|---|---|
| Fresh Beef ... ... ... ... | 35 to 40 |
| Fresh Lamb and Mutton ... ... | 35 to 40 |
| Frozen Lamb and Mutton ... ... | 25 to 30 |
| Frozen Poultry ... ... ... | 28 to 30 |
| Fresh Fish ... ... ... ... | 25 to 30 |
| Dried Fish ... ... ... ... | 35 |
| Cheese ... ... ... ... ... | 28 to 45 |
| Beer ... ... ... ... ... | 33 to 45 |
| Butter and Margarine ... ... ... | 25 to 35 |

The cold store, though dry, must not be too dry, as it would then favour the shrinkage of the stores.

If the moisture is kept (as before stated) below the point of saturation, the best results are obtained, but efficient ventilation will safely regulate this condition.

The air should always be taken in at the ceiling level, or near it, to ensure a continual movement in the chamber.

There are two ammonia systems now in use—

namely, the absorption and compression systems. It is very generally conceded that the compression system for practical purposes is by far the better. The medium used as the refrigerant is anhydrous ammonia. There are other systems using other refrigerants than ammonia, such as carbon dioxide, sulphur dioxide and air, but there are so few machines of this type in actual operation that they need scarcely be considered.

Anhydrous ammonia is a volatile liquid, by the evaporation of which cold is produced; it boils at about 28½° below zero Fahrenheit when under atmospheric pressure. At lower temperatures it is a liquid, while at higher temperatures, and this includes the ordinary range of storage temperature, it is a gas. It is this low boiling-point which enhances its value as a refrigerating agent. When allowed to evaporate, it takes up heat in the process, and every pound of ammonia is capable of taking up a certain quantity of heat. Ammonia being an expensive product, part of the refrigerating plant, called the compressor, is designed to economise its use, but of this hereafter.

There are three processes necessary in the compression system of refrigeration, *viz.* compression, condensation, and expansion of the cold producing agent, which three form a continuous cycle. These three processes are accomplished by machinery and appliances adapted for their particular duties. The compressor is a type of pump designed for the purpose of compressing the ammonia gas, and forcing it into the condenser under pressure. The condenser is a series of coils of special welded tube, inside which the compressed vapours are cooled and liquefied by a constant external flow of water, either by complete immersion in a wrought-iron tank or by a series of sprays.

The refrigerator or expansion coils are a series of special welded tubings wound each in one length, so as to avoid inaccessible joints. Into these coils the liquid ammonia is allowed to expand through a small orifice in the expansion valve; this liquid expands to about 1,500 times its volume, and in so doing absorbs heat from the surrounding atmosphere or agents in contact, as the case may be, thus producing intense cold.

The expansion coils are connected to the suction pipe leading back to the compressor, and thus the gas returns to renew its cycle. From the above it will be seen that the heat absorbed by the ammonia is in its turn absorbed by the cold water circulating through the condenser. The ammonia then is simply the agent by which a comparatively high temperature in the water produces a low temperature in the rooms, or boxes, to be cooled.

The ammonia is not used up in the process of refrigeration, but travels from the condenser and compressor to the expansion coils, and is then pumped back in a continuous cycle. (See illustration.) The expense for ammonia is limited to that which escapes from the system through leakage and will depend on the care and attention given by the operator, and further on the type of machine employed.

DIAGRAM SHOWING PRINCIPLE OF Dr LINDE'S SYSTEM OF REFRIGERATION.

(*To be continued.*)

## Figures and Tables

**Figure f1:**